# Prevalence and correlates of hyperglycemia in a rural population, Vietnam: implications from a cross–sectional study

**DOI:** 10.1186/1471-2458-12-939

**Published:** 2012-11-01

**Authors:** Tran Quang Binh, Pham Tran Phuong, Bui Thi Nhung, Dang Dinh Thoang, Pham Van Thang, Tran Khanh Long, Duong Van Thanh

**Affiliations:** 1 Department of Immunology and Molecular Biology, National Institute of Hygiene and Epidemiology, 1 Yersin, Hanoi, Vietnam; 2 National Institute of Nutrition, 48B Tang Bat Ho Street, Hanoi, Vietnam; 3 Ha Nam Center for Preventive Medicine, Truong Chinh Street, Phu Ly City, Vietnam; 4 Hanoi School of Public Health, 138 Giang Vo Street, Hanoi, Vietnam

**Keywords:** Type 2 diabetes, Population–based study, Prevalence, Risk factors, Vietnamese

## Abstract

**Background:**

Despite the increasing prevalence of type 2 diabetes in urban areas, relatively little has been known about its actual prevalence and its associations in rural areas, Vietnam. The purpose of this study was to evaluate the prevalence of impaired fasting glucose (IFG), impaired glucose tolerance (IGT), diabetes and their risk factors in a rural province, Vietnam.

**Methods:**

A cross–sectional study with a representative sample was designed to estimate the hyperglycemia prevalence, using 75–g oral glucose tolerance test. Potential risk factors for hyperglycemia were analyzed using multinomial logistic regression, taken into account influences of socio–economic status, anthropometric measures, and lifestyle–related factors.

**Results:**

The age and sex–adjusted prevalence rates (95% CI) of isolated IFG, isolated IGT, combined IFG–IGT, and diabetes were 8.7 (7.0–10.5), 4.3 (3.2−5.4), 1.6 (0.9−2.3), and 3.7% (2.7–4.7%), respectively. There were still 73% of diabetic subjects without knowing the condition. Blood pressure, family history of diabetes, obesity–related measures (waist circumference, waist–hip ratio, body fat percentage, and abdominal obesity) were the independent risk factors for hyperglycemia (IFG, IGT, and diabetes).

**Conclusions:**

The prevalence of hyperglycemia in rural areas has not been as sharply increased as that reported in urban cities, Vietnam. Blood pressure and obesity–related measures were the most significant predictors for hyperglycemia level and they can be taken into account in building prognosis models to early detection of diabetes in rural Vietnamese populations.

## Background

Type 2 diabetes, also known as adult onset or non–insulin dependent diabetes, has become a global public health problem. It is estimated that the world prevalence of diabetes among adults aged 20–79 years will increase to 7.7%, affecting 439 million adults by the year 2030. There will be a 69% increase in numbers of adults with diabetes in developing countries between 2010 and 2030 [1]. In Vietnam, the prevalence of type 2 diabetes in Hanoi increased from 1.4% in 1990 to 4.4% in 2002 in residents aged 30−64 years [2,3]. In Ho Chi Minh City, the residents aged 15 years or over had a 6.6% prevalence rate of type 2 diabetes in 2001, a significant increase from 2.5% in 1993; and the prevalence of the disease in residents aged 30−72 years reached 11.4% in 2009 [4-6]. The number of Vietnamese diabetic cases will be double in 2030 compared to the year 2010 [1]. In order to evaluate the burden of diabetes and implement an effective strategy for prevention of diabetes, it is crucial to understand the prevalence and risk factors of the disease in both urban and rural populations.

Despite the worldwide importance of diabetes, relatively little has been known about its actual prevalence and its associations in Vietnam. The epidemiology studies of diabetes in Vietnam mainly focused on big cities where the burden of diabetes was thought to be high due to industrialization and the shift of dietary habits to a high fat intake [7]. However, there has been a limited data on the hyperglycemia status and related risk factors in rural areas with more than 71 % of the total population in Vietnam [8]. Therefore, we conducted a cross–sectional study to identify the prevalence of impaired fasting glucose, impaired glucose tolerance, type 2 diabetes, and related risk factors in rural Vietnamese population.

## Methods

### Setting and study subjects

The study was designed as a cross–sectional investigation, conducted in Ha Nam province, Vietnam from July to November 2011. Located in the south–west of the Red River Delta and 50 km far from Hanoi Capital, Ha Nam province has a population of approximately 800,000 inhabitants, living mostly in rural areas (108 rural communes and 6 urban wards) [5]. The Ethics Committee of the National Institute of Hygiene and Epidemiology, Vietnam approved the protocol of the survey. All participants provided written informed consent before entering the study.

The sample size was calculated to estimate the prevalence of type 2 diabetes of 5.5% within 0.012 with 95% confidence interval, considering the following parameters: α = 0.05, β = 0.2, a design effect = 2 for multistage stratified random study, and non−response rate = 8%. The two–stage sampling method was used to recruit 3,000 subjects aged 40–64 years. First, using the probability proportion–to–size method, we selected 30 of the all 114 communes and wards in Ha Nam province. A listing of all local persons aged 40–64 years was constructed in each of the selected communes and wards. From this list, the simple random sampling was used to recruit 100 unrelated participants for the study. Exclusion criteria for potential participants included pregnant women, critically ill subjects, and mentally disordered subjects. To maximize participation, the local health staff visited each household to explain the study, obtain written consent from each subject, and remind participants of the time and date of the survey. In the days of survey, 290 (9.7%) subjects were absent or excluded due to ages < 40 or > 64 years. As a results, 2,710 subjects participated into the study.

### Diagnosis of type 2 diabetes

All participants were instructed to fast overnight before the day of survey. Blood samples were collected and centrifuged immediately in the morning after a participant had fasted for at least 8 h prior to the clinic visit. Aliquots of plasma were stored at 2–8°C in iceboxes and then transported into the central laboratory of the Ha Nam Center for Preventive Medicine for analysis within 6 hours. Plasma glucose was measured by glucose oxidase method (GOD–PAP), using a semi–autoanalyzer (Screen Master Lab; Hospitex Diagnostics LIHD112, Italy) with commercial kit (Chema. Diagnostica, Italy).

The World Health Organization and International Diabetes Federation diagnostic criteria of type 2 diabetes was used to determine the glycaemic status of subjects [9], using fasting plasma glucose level (FPG) and oral glucose tolerance test (OGTT) with 75 gram glucose. All participants, except for those having previous diagnosis of diabetes and current use of drug for its treatment, underwent an OGTT test. A participant was classified as having diabetes as FPG ≥ 7.0 mmol/l or 2 h plasma glucose ≥ 11.1 mmol/l or previous diagnosis of diabetes and current use of drug for its treatment. Normal glucose tolerance (NGT) was classified when FPG < 5.6 mmol/l and 2 h plasma glucose < 7.8 mmol/l. Isolated impaired fasting glucose (IFG) was identified if FPG was between 5.6 and 6.9 mmol/l, and 2 h plasma glucose was less than 7.8 mmol/l. Isolated impaired glucose tolerance (IGT) was classified if FPG was less than 5.6 mmol/l and 2 h plasma glucose was between 7.8 and 11.0 mmol/l. Combined IFG and IGT (IFG−IGT) were determined if FPG was between 5.6 and 6.9 mmol/l, and 2 h plasma glucose was between 7.8 and 11.0 mmol/l.

### Data collection

All participants were interviewed by trained surveyors to complete a structured questionnaire. Data were collected on current age, ethnicity, educational level, occupation, family history of diabetes, medical and reproductive history, smoking and drinking history, time spent for night’s sleep, siesta, and watching television (TV).

Individuals were classified as having a family history of diabetes if they reported that any first–degree relative (parent or sibling) suffered from diabetes. Lifelong occupation was defined as the occupation that the subject engaged in most frequently in the life. It was categorized as heavy occupation (farmer and manual worker) and none heavy occupation (office clerks, teacher, retired worker, and houseworker). Educational level was categorized in four groups, by number of years of schooling: elementary level (≤ 5 years), intermediate level (6–9 years), secondary level (10–12 years), and post–secondary level (> 12 years). Usual alcohol consumption over the past year was assessed via an in–person interview. For each beverage type (beer, wine), participants reported their consumption frequency and portion size. These data were combined to yield 4 alcohol consumption categories: none (never or < 1 drink/mo), ≥ 1 drink/mo to < 1 drink/wk, 1 drink/wk to ≤ 1 drink/d, and ≥ 2 drink/d, in which one drink was defined as a 50–ml cup of rice wine at about 30%.

### Anthropometric and clinical measurements

Weight, height, waist and hip circumference, body fat percentage were measured twice in each individual and the mean was used for the purpose of analysis. Body weight and height were measured in light clothing and without shoes. Body mass index (BMI) was calculated as weight per square of height (kg/m^2^). Waist circumference (WC) was measured at the mid–way between the lower rib margin and the iliac crest, while hip circumference (HC) was measured at the broadest circumference around the buttocks. Waist–hip ratio (WHR) was calculated as waist circumference (cm) divided by hip circumference (cm). Body fat percentage was measured by bioelectrical impedance method by using OMRON scale (HBF–351, Kyoto, Japan). Overweight and obesity were classified by the BMI–value recommendations of the World Health Organization [10] and the International Obesity Task Force for Asian and Pacific Island populations, which corresponds to the BMI cutoffs of 23 and 25 kg/m^2^[11]. Blood pressure was measured twice in a sitting position after participants rested for at least 5 min. The mean of the two values was used in the analysis. High blood pressure was defined as a systolic blood pressure (SBP) of at least 130 mmHg and/or a diastolic blood pressure (DBP) of at least 85 mmHg.

### Statistical analysis

Data were weighted, taken into account the study design, the probability of sampling, finite population correction, and none–response rate. The estimated prevalences of isolated IFG, isolated IGT, combined IFG−IGT, and diabetes were computed for the whole Ha Nam population aged 40–64 years together with subgroups according to age group, sex, nutritional status, and residence. The age and sex–adjusted prevalences were estimated using direct standardization method based on the 2009 Vietnam Population and Housing Census [8].

Quantitative variables were checked for normal distribution and compared using One–Way ANOVA or Independent–Sample T test. Kruskal–Wallis or Mann–Whitney U test was used to compare quantitative variables without normal distribution. Frequencies of category variables were compared by Pearson’s χ^2^ test or Fisher’s exact test when appropriate. Univariate logistic regression analysis was used to assess potential factors associated with IFG, IGT, IFG−IGT, and diabetes. Multinomial logistic regression analysis with backward stepwise method was performed to test several models for the associations of high blood glucose levels to the potential risk factors including: i) socio–economic conditions: age, sex, residence, occupation, marital status, income level, education level, family history of diabetes; ii) anthropometric and clinical measures: weight, BMI, body fat percentage, waist circumference, waist–hip ratio, abdominal obesity, nutrition status, blood pressure; and iii) lifestyle−related factors: alcohol consumption, smoking, time spending for night’s sleep, siesta, and watching TV. Here, data are presented as odds ratios with 95 percent confidence intervals (CI). Associations were considered statistically significant at two–sided *P* values of less than 0.05 for all the analyses. The above statistical procedures were performed using Stata version 11.2 and SPSS version 16.0.

## Results

### Characteristics of the study cohort

Of the 2,710 participants attending the study, 65% were women, 72% were farmers, and 72% had elementary and intermediate levels of education. Table 1 presents the characteristics of the subjects according to blood glucose levels. SBP and DBP were significantly lower in NGT group in comparison with IFG, IGT, and diabetes groups. Except for height, there was a trend of stepwise increase in obesity–related traits including weight, BMI, WC, HC, WHR, and body fat percentage between NGT, IFG, IGT and diabetes groups. Diabetic subjects had significantly higher values of the obesity–related traits than those with NGT. Subjects with IGT had much higher values of body fat percentage, WC, WHR, and BMI (in men) than those with NGT. There were not significant differences in anthropometric measures among NGT, IFG, and combined IFG−IGT groups. The body fat percentage was markedly higher in women compared to men (*P* < 0.0001, Student–T test) in all blood glucose levels, whereas there was no statistically significant difference in BMI between men and women.

**Table 1:** Characteristics of the studied subjects according to blood glucose levels in Ha Nam province, 2011

**Variables**	**Total (*n*= 2,710)**	**NGT (*n*= 2,199)**	**IFG (*n*= 248)**	**IGT (*n*= 120)**	**IFG−IGT (*n*= 43)**	**Diabetes (*n*= 100)**
Age (year)†	51 (46–56)	51 (46–56)	52 (47–58)**	53 (48–58)**	54 (46–57)	54 (49–60)***
SBP (mmHg)†	115 (100–130)	110 (100–128)	120 (110–136)***	120 (110–138)***	110 (100–140)	124 (110–140)***
DBP (mmHg)†	70 (65–80)	70 (65–80)	80 (70–81)***	80 (70–90)***	70 (60–80)	80 (70–88)***
Height (cm)	155.7 ± 7.0	155.6 ± 6.9	155.9 ± 6.8	154.9 ± 7.6	154.0 ± 6.8	156.9 ± 7.3
Weight (kg)	51.8 ± 8.0	51.5 ± 7.8	51.6 ± 7.9	53.3 ± 8.8	52.8 ± 7.4	55.8 ± 10.4***
BMI (kg/m^2^)						
Men	21.3 ± 2.7	21.1 ± 2.6	21.4 ± 2.8	22.8 ± 2.8**	22.9 ± 2.0*	22.4 ± 3.4*
Women	21.4 ± 2.7	21.3 ± 2.6	21.0 ± 2.5	21.7 ± 2.5	21.9 ± 2.4	22.7 ± 3.5***
Body fat (%)						
Men	21.8 ± 5.4	21.5 ± 5.4	22.4 ± 5.2	24.4 ± 4.6**	23.3 ± 3.8	23.4 ± 6.2
Women	30.0 ± 4.6	29.9 ± 4.6	29.6 ± 4.5	31.4 ± 4.3*	31.2 ± 3.4	33.1 ± 4.5***
WC (cm)	74.4 ± 7.8	73.9 ± 7.6	74.5 ± 7.9	77.1 ± 8.6**	75.3 ± 6.5	79.8 ± 9.5***
HC (cm)	87.9 ± 5.6	87.9 ± 5.5	87.5 ± 5.9	88.8 ± 6.0	88.4 ± 4.7	90.3 ± 6.3***
WHR						
Men	0.86 ± 0.06	0.86 ± 0.06	0.88 ± 0.10	0.89 ± 0.06**	0.88 ± 0.03	0.90 ± 0.07**
Women	0.84 ± 0.06	0.83 ± 0.06	0.84 ± 0.06	0.85 ± 0.06*	0.84 ± 0.05	0.87 ± 0.06***

*NGT*, normal glucose tolerance; *IFG*, isolated impaired fasting glucose; *IGT*, isolated impaired glucose tolerance; *BMI*, body mass index; *WC*, waist circumference; *HC*, hip circumference; *WHR*, waist–hip ratio; *SBP*, systolic blood pressure; *DBP*, diastolic blood pressure.

Data are mean ± SD unless otherwise indicated. †Data are median (intequartile range).

* *P* < 0.05, ** *P* < 0.01, *** *P* < 0.001: NGT group vs. hyperglycemia groups by Student–T test or one–way ANOVA test or Mann–Whitney U test.

### Prevalence of hyperglycemia

The crude prevalences of isolated IFG, isolated IGT, combined IFG−IGT, and diabetes were 9.2 (8.1−10.2), 4.4 (3.7−5.2), 1.6 (1.1−2.1), and 3.7% (3.0−4.4%), respectively. The total age and sex–adjusted prevalences (95% CI) of isolated IFG, isolated IGT, combined IFG–IGT, and diabetes were 8.7 (7.0–10.5), 4.3 (3.2−5.4), 1.6 (0.9−2.3), and 3.7% (2.7–4.7%), respectively. The number of newly diagnosed cases occupied 73% of the total diabetic patients. Without relying on the OGTT test, we would have missed 40% of the total diabetic cases.

Table 2 shows the estimated prevalence of hyperglycemia in people aged 40–64 years in Ha Nam province. The estimated prevalences of IFG, IGT, and diabetes were notable higher in men, without heavy jobs, and urban area, in comparison with women, with heavy jobs, and rural area, respectively. The estimated prevalence of diabetes increased with age and reached a peak in the oldest group. The prevalences of IGT and diabetes were significantly higher in the obese group compared to normal group. The underweight status was not found to be significantly associated with IGT, IFG−IGT, and diabetes.

**Table 2:** Estimated prevalence of IFG, IGT, IFG−IGT, and diabetes in Vietnamese adults aged 40−64 years in Ha Nam province, 2011

**Variables**	**IFG**	**IGT**	**IFG−IGT**	**Diabetes**		**Previous**
				**Total**	**New**	
Age group (year)						
40−44	6.9 (5.9−7.9)	3.0 (2.2−3.7)	1.5 (1.0−2.0)	2.4 (1.8−3.0)	1.7 (1.2−2.3)	0.7 (0.3−1.0)
45−49	7.4 (6.1−8.62)	3.2 (2.5−3.9)	1.3 (0.9−1.7)	2.5 (2.0−3.0)	1.8 (1.4−2.2)	0.7 (0.4−0.9)
50−54	9.5 (8.2−10.9)	5.3 (4.5−6.0)	1.2 (0.8−1.5)	3.4 (2.9−4.0)	2.2 (1.8−2.7)	1.2 (0.9−1.6)
55−59	11.5 (10.0−12.9)	5.5 (4.4−6.5)	3.3 (2.6−3.9)	4.6 (3.9−5.4)	3.5 (2.7−4.3)	1.1 (0.7−1.6)
60−64	11.0 (9.2−12.7)	5.3 (4.2−6.4)	0.9 (0.5−1.3)	6.4 (5.0−7.7)	5.2 (4.1−6.3)	1.2 (0.7−1.7)
Adjusted total†	8.7 (7.0−10.5)	4.3 (3.2−5.4)	1.6 (0.9–2.3)	3.7 (2.7–4.7)	2.7 (1.8−3.5)	0.8 (0.3−1.4)
Men						
40−44	7.0 (4.9−9.0)	4.8 (3.2−6.3)	2.3 (1.3−3.4)	5.2 (3.5−7.0)	3.3 (1.9−4.7)	1.9 (0.7−3.1)
45−49	7.0 (5.4−8.5)	1.3 (0.7−2.0)	1.3 (0.7−2.1)	4.0 (2.9−5.1)	2.9 (1.9−4.0)	1.0 (0.5−1.5)
50−54	10.5 (8.8−12.1)	7.3 (5.9−8.6)	1.2 (0.5−1.9)	3.4 (2.6−4.3)	2.0 (1.3−2.8)	1.4 (0.8−1.9)
55−59	13.6 (11.7−15.6)	5.7 (4.1−7.3)	1.7 (0.9−2.5)	5.3 (4.0−6.6)	4.0 (2.8−5.3)	1.2 (0.6−1.8)
60−64	9.0 (6.3−11.7)	5.8 (3.8−7.8)	0.7 (0.1−1.2)	6.0 (4.3−7.8)	4.4 (2.8−5.9)	1.6 (0.8−2.5)
Adjusted total†	8.9 (7.1−10.8)	4.8 (3.5−6.0)	1.5 (0.8−2.3)	4.6 (3.3−5.8)	3.1 (2.0−4.2)	1.0 (0.3−1.7)
Women						
40−44	6.9 (5.7−8.1)	2.4 (1.6−3.1)	1.3 (0.7−1.8)	1.4 (1.0−1.9	1.2 (0.7−1.7)	0.2 (0.1−0.4)
45−49	7.6 (6.1−9.0)	4.0 (3.1−5.0)	1.3 (0.7−1.8)	1.8 (1.3−2.3)	1.3 (0.9−1.7)	0.5 (0.2−0.8)
50−54	9.0 (7.0−11.0)	4.1 (3.3−4.9)	1.1 (0.7−1.5)	3.4 (2.7−4.2)	2.3 (1.7−2.9)	1.1 (0.7−1.6)
55−59	10.1 (8.3−11.9)	5.3 (4.2−6.4)	4.3 (3.3−5.3)	4.2 (3.3−5.1)	3.2 (2.3−4.1)	1.1 (0.6−1.6)
60−64	12.5 (10.0−14.9)	4.9 (3.6−6.3)	1.0 (0.4−1.6)	6.6 (5.0−8.3)	5.8 (4.4−7.3)	0.8 (0.3−1.3)
Adjusted total†	8.6 (7.0−10.2)	3.9 (3.0−4.9)	1.7 (1.1−2.3)	2.9 (2.2−3.7)	2.3 (1.6−2.9)	0.7 (0.3−1.0)
Residence						
Rural	8.4 (7.6−9.3)	4.0 (3.5−4.4)	1.4 (1.2−1.7)	3.5 (3.1−3.8)	2.5 (2.2−2.8)	1.0 (0.9−1.2)
Urban	16.5 (12−21)	9.0 (7.8−10.2)	3.9 (1.5−6.3)	5.5 (3.9−7.1)	4.7 (2.7−6.7)	0.8 (0.3−1.3)
Occupation						
Heavy job	9.3 (8.2−10.3)	4.0 (3.5−4.5)	1.5 (1.3−1.7)	3.0 (2.7−3.3)	2.4 (2.1−2.7)	0.6 (0.5−0.8)
None heavy job	8.5 (7.4−9.6)	5.7 (4.8−6.7)	2.2 (1.3−3.1)	6.1 (5.2−6.9)	3.9 (3.2−4.7)	2.2 (1.6−2.8)
Nutrition status						
Normal	8.4 (7.5−9.3)	4.5 (3.9−5.1)	1.7 (1.3−2.0)	2.8 (2.4−3.2)	2.0 (1.7−2.3)	0.8 (0.6−0.9)
Overweight	11.1 (9.5−12.6)	4.6 (3.8−5.4)	2.0 (1.3−2.6)	4.7 (3.8−5.5)	3.4 (2.6−4.1)	1.3 (0.9−1.8)
Obesity	7.8 (6.3−9.3)	7.0 (5.8−8.2)	2.6 (1.7−3.6)	8.7 (6.6−10.7)	7.1 (5.3−9.0)	1.5 (0.5−2.5)
Underweight	11.3 (9.5−13.1)	2.1 (1.5−2.6)	0.5 (0.2−0.8)	2.9 (2.1−3.6)	2.0 (1.4−2.5)	0.9 (0.4−1.4)

*NGT*, normal glucose tolerance; *IFG*, isolated impaired fasting glucose; *IGT*, isolated impaired glucose tolerance; *IGF−IGT*, combined IFG and IGT. Data are presented as % (95%CI). Occupation was categorized as heavy occupation (farmer and manual worker) or none heavy occupation (office clerks, teacher, retired worker, and houseworker). Overweight was defined as BMI ≥ 23 kg/m^2^. Obesity was defined as BMI ≥ 25 kg/m^2^. †Age and sex adjustment based on the 2009 Vietnam Population and Housing Census using direct standardization method.

### Risk factors associated with hyperglycemia levels

The univariate logistic regression analysis showed the potential factors associated with impaired glucose homeostasis levels: age, sex, blood pressure, nutritional status, obesity–related traits, family history of diabetes, residence, occupation, alcohol consumption, smoking, time spent for night’s sleep, siesta, and watching TV were statistically significantly associated with diabetes; marital status, income level, education level, and smoking status were not significantly associated with diabetes, IFG, and IGT (Additional file 1).

Table 3 presents the factors associated with IFG, IGT, and diabetes in multinomial logistic regression models adjusted for socio–economic conditions, anthropometric and clinical measures, and lifestyle−related factors. The initial analysis showed that blood pressure, waist−hip ratio, and family history of diabetes were the most significantly associated with diabetes; blood pressure, marital status, and residence were associated with IGT and IFG−IGT; and blood pressure, waist-hip ratio, residence, and alcohol consumption were associated with IFG. The final result from the backward stepwise method to remove non-significant variables from the models showed that family history of diabetes, blood pressure, waist−hip ratio, body fat percentage, residence, and alcohol consumption remained positively associated with hyperglycemia levels (Table 4). The same model in Table 4 with replacing waist-hip ratio by other obesity-related traits including waist circumference or abdominal obesity showed that waist circumference (OR = 1.08, 95%CI=1.04−1.12, *P* < 0.0001) and abdominal obesity (OR = 2.41, 95%CI=1.41−4.11, *P* = 0.001) were also significantly associated with diabetes.

**Table 3:** Associated factors of IFG, IGT, and diabetes in multinomial logistic regression analysis

**Variables**	**IFG (*n*= 248)**		**IGT and IGT−IFG (*n*= 163)**		**Diabetes (*n*= 100)**	
	**OR (95%CI)**	***P***	**OR (95%CI)**	***P***	**OR**	***P***
Sex						
Women	1		1		1	
Men	0.60 (0.32−1.15)	0.122	1.09 (0.51−2.33)	0.819	1.65 (0.70−3.92)	0.253
Age (year)	1.02 (0.99−1.05)	0.057	1.01 (0.98−1.04)	0.590	1.04 (1.00−1.08)	0.076
Blood pressure						
Normal	1		1		1	
High	1.66 (1.24−2.24)	0.001	1.68 (1.18−2.40)	0.004	1.71 (1.10−2.67)	0.018
BMI (kg/m^2^)	0.95 (0.89−1.03)	0.202	1.03 (0.95−1.12)	0.479	1.05 (0.95−1.16)	0.371
Body fat (%)	0.99 (0.95−1.03)	0.493	1.03 (0.99−1.08)	0.162	1.03 (0.98−1.10)	0.252
Waist-hip ratio (per SD=0.07)	1.21 (1.01−1.46)	0.048	1.21 (0.97−1.52)	0.094	1.47 (1.14−1.90)	0.003
Family history of diabetes						
No	1		1		1	
Yes	1.44 (0.73−2.82)	0.290	2.00 (0.98−4.06)	0.056	4.24 (2.10−8.57)	<0.0001
Marital status						
Married	1		1		1	
Never	1.96 (0.83−4.63)	0.126	2.69 (1.05−6.86)	0.039	2.26 (0.64−7.99)	0.207
Widowed	1.30 (0.74−2.27)	0.357	0.96 (0.46−2.02)	0.921	1.66 (0.71−3.88)	0.240
Others	0.95 (0.28−3.17)	0.930	1.02 (0.24−4.43)	0.977	1.06 (0.14−8.08)	0.958
Education level						
Elementary	1		1		1	
Intermediate	1.43 (0.86−2.37)	0.172	0.62 (0.37−1.04)	0.069	1.01 (0.45−2.24)	0.986
Secondary	1.16 (0.61−2.24)	0.649	0.68 (0.34−1.36)	0.270	1.53 (0.59−4.00)	0.385
Post–secondary	1.09 (0.55−2.19)	0.803	0.60 (0.29−1.27)	0.182	1.18 (0.44−3.21)	0.742
Residence						
Rural	1		1		1	
Urban	3.00 (1.83−4.90)	<0.0001	3.22 (1.85−5.60)	<0.0001	1.53 (0.70−3.34)	0.289
Heavy occupation						
Yes	1		1		1	
No	0.86 (0.55−1.34)	0.504	1.17 (0.71−1.92)	0.538	1.41 (0.79−2.51)	0.251
Income level						
< 25 percentiles	1		1		1	
25–<50 percentiles	1.17 (0.78−1.73)	0.449	1.69 (1.06−2.70)	0.028	0.85 (0.46−1.58)	0.605
50–<75 percentiles	1.18 (0.79−1.77)	0.427	0.97 (0.57−1.65)	0.899	0.86 (0.46−1.60)	0.629
≥75 percentiles	1.34 (0.89−2.01)	0.163	1.00 (0.59−1.70)	0.994	0.74 (0.40−1.39)	0.353
Alcohol consumption						
None	1		1		1	
<1 drink/mo	1.28 (0.72−2.29)	0.398	1.24 (0.61−2.50)	0.555	1.13 (0.46−2.78)	0.794
≥ 1 drink/mo to < 1 drink/wk	2.03 (1.15−3.61)	0.015	1.84 (0.92−3.66)	0.084	1.33 (0.53−3.35)	0.545
1 drink/wk to ≤ 1 drink/d	2.40 (1.40−4.13)	0.001	0.88 (0.43−1.77)	0.715	0.63 (0.26−1.49)	0.292
≥ 2 drink/d	2.29 (1.23−4.25)	0.009	1.18 (0.57−2.45)	0.652	1.41 (0.63−3.17)	0.408
Smoking						
None	1		1		1	
Current smoker	0.66 (0.38−1.16)	0.145	0.96 (0.46−2.01)	0.920	0.82 (0.36−1.86)	0.638
Ex–smoker	0.83 (0.46−1.50)	0.530	1.35 (0.65−2.81)	0.430	0.86 (0.38−1.95)	0.713
Watching TV time/day						
≤ 3 hours	1		1		1	
> 3 hours	0.90 (0.46−1.76)	0.757	0.73 (0.32−1.66)	0.449	1.29 (0.60−2.78)	0.514
Siesta time/day						
None	1		1		1	
<30 min	1.29 (0.81−2.06)	0.281	1.06 (0.61−1.84)	0.844	1.82 (0.80−4.12)	0.154
30–<60 min	1.08 (0.65−1.79)	0.774	0.93 (0.51−1.69)	0.812	1.43 (0.60−3.42)	0.421
60–<90 min	1.43 (0.86−2.38)	0.164	1.26 (0.69−2.28)	0.455	1.70 (0.71−4.05)	0.234
≥ 90 min	1.28 (0.66−2.49)	0.473	1.63 (0.78−3.39)	0.191	2.30 (0.86−6.18)	0.098
Sitting time/day						
≤ 4 hours	1		1		1	
> 4 hours	0.91 (0.67−1.23)	0.533	1.06 (0.74−1.54)	0.744	1.43 (0.91−2.26)	0.124

*IFG*, isolated impaired fasting glucose; *IGT*, isolated impaired glucose tolerance; *IGF−IGT*, combined IFG and IGT.

High blood pressure: systolic blood pressure ≥ 130 mmHg and/or diastolic blood pressure ≥ 85 mmHg.

Educational level was categorized in four groups, by number of years of schooling: elementary level (≤ 5 years), intermediate level (6–9 years), secondary level (10–12 years), and post–secondary level (> 12 years). Occupation was categorized as heavy occupation (farmer and manual worker) or none heavy occupation (office clerks, teacher, retired worker, and house worker).

OR and *P* values were adjusted by all variables in the table.

**Table 4:** Associated factors of hyperglycemia levels in multinomial logistic regression with backward stepwise method

**Variables**	**IFG (*n*= 248)**		**IGT and IGT−IFG (*n*= 163)**		**Diabetes (*n*= 100)**	
	**OR (95% CI)**	***P***	**OR (95% CI)**	***P***	**OR (95% CI)**	***P***
Residence						
Rural	1		1		1	
Urban	2.68 (1.68-4.27)	< 0.0001	2.88 (1.71-4.85)	< 0.0001	1.63 (0.78-3.43)	0.197
Family history of diabetes						
No	1		1		1	
Yes	1.38 (0.71−2.68)	0.347	1.93 (0.96−3.89)	0.067	4.44 (2.26−8.75)	< 0.0001
Blood pressure						
Normal	1		1		1	
High	1.66 (1.24−2.22)	0.001	1.71 (1.21−2.43)	0.003	1.77 (1.15−2.74)	0.010
Waist-hip ratio (per SD = 0.07)	1.17 (0.98−1.40)	0.082	1.25 (1.01−1.53)	0.037	1.56 (1.24−1.95)	< 0.0001
Body fat (%)	0.97 (0.94−1.00)	0.083	1.05 (1.01−1.09)	0.029	1.05 (1.01−1.10)	0.043
Alcohol consumption						
None	1		1		1	
<1 drink/mo	1.27 (0.72−2.24)	0.416	1.20 (0.60−2.37)	0.607	1.18 (0.49−2.84)	0.714
≥ 1 drink/mo to < 1 drink/wk	1.98 (1.12−3.49)	0.019	1.71 (0.88−3.33)	0.117	1.30 (0.53−3.21)	0.571
1 drink/wk to ≤ 1 drink/d	2.30 (1.34−3.94)	0.002	0.88 (0.44−1.74)	0.707	0.66 (0.28−1.55)	0.340
≥ 2 drink/d	2.14 (1.17−3.91)	0.014	1.13 (0.56−2.27)	0.728	1.49 (0.69−3.19)	0.312

*IFG*, isolated impaired fasting glucose; *IGT*, isolated impaired glucose tolerance; *IGF−IGT*, combined IFG and IGT.

High blood pressure: systolic blood pressure ≥ 130 mmHg and/or diastolic blood pressure ≥ 85 mmHg.

OR and *P* values were adjusted by age and sex.

## Discussion

Despite the worldwide importance of diabetes, relatively little has been known about its actual prevalence and its associations in Vietnam, especially in rural areas. It is the first report on the status of impaired glucose homeostasis and its risk factors in a rural province of Northern Vietnam. The results of the representative, population–based study showed that the age and sex–adjusted prevalences (95% CI) of isolated IFG, isolated IGT, combined IFG−IGT and diabetes in Ha Nam province were 8.7 (7.0–10.5), 4.3 (3.2−5.4), 1.6 (0.9−2.3), and 3.7% (2.7–4.7%), respectively. These age–standardized prevalences of IGT and diabetes were lower than those reported in plain region in the 2002 national survey of diabetes, whereas the prevalence of IFG was much higher with the same diagnosis criteria [3]. Moreover, these prevalences in the rural province were much lower than those in urban cities in Vietnam [3,6]. In comparison with other rural populations in Asia, our study reported lower prevalences of diabetes and IGT than those reported in Central India [12], in Guangdong province of China [13], in Thailand [14], in Japan [15], and in Korea [16]. Fortunately, the present study indicates that the prevalence of hyperglycemia in rural areas has not been as sharply increased as that reported in urban cities, Vietnam. It could be explained by the traditional lifestyle may remain conservative in rural areas and by the “nutrition transition” [17] keeps low stage in this process with 11% underweight subjects (BMI < 18.5 kg/m^2^) and 7.7% obese subjects (BMI ≥ 25 kg/m^2^).

The estimated prevalence of IFG and IGT increased in 50−54 age group compared to 40−49 age group, and the prevalence was not different between 50−59 age group and 60−64 age group. The prevalence of combined IFG−IGT was significantly higher in group aged 50−59 years compared to the others. The findings indicated that prevalence of prediabetes did not show age effect, consistent with other reports [18,19]. Our results can be explained by: i) 5−10% of people per year with prediabetes may progress to diabetes, with the same proportion converting back to normoglycaemia [20], and ii) people with prediabetes may postpone or completely avoid the onset of type 2 diabetes with three simple strategies including losing weight, increasing physical activity, and eating more healthfully [20].

Because diabetes are known as multifactor and lifestyle–related disorders, we took into account the analysis of potential risk factors, including: i) socio–economic conditions: age, sex, residence, occupation, marital status, income level, education level, and family history of diabetes; ii) anthropometric and clinical measures: weight, BMI, body fat percentage, waist circumference, hip circumference, waist–hip ratio, abdominal obesity, nutrition status, and blood pressure; and iii) lifestyle factors: alcohol consumption, smoking, time spending for night’s sleep, siesta, and watching TV. As a result, high blood pressure, obesity-related measures (waist-hip ratio, body fat percentage, WC, and abdominal obesity), and family history of diabetes were the most significantly associated with diabetes. The association was observed in the univariate analysis, confirmed in multinomial logistic regression model adjusted, and remained in the analysis with backward stepwise method to remove non-significant variables from models. However, there were 73% of diabetic subjects without knowing the condition, suggesting the major part of people in the community do not know the real status of their family history of diabetes. Thus, using the family history of diabetes in the predict models for diabetes should be considered. Taken together, blood pressure and obesity–related measures appear to be the most significant predictors for hyperglycemia level and they should be taken into account in building prognosis models to early detection of diabetes in rural Vietnamese populations.

With regard to the relationship between the elevated blood pressure and diabetes, both disorders commonly occur together and tend to share many predisposing factors including obesity, physical inactivity, and high−fat diets [21,22]. Each disease tends to affect patients who are already at risk for the other. Recent studies suggest that the elevated blood pressure may also precede the type 2 diabetes [23,24]. The elevated blood sugar has many consequences, including slow but serious damage to sensitive capillaries in the kidneys. This damage impairs the kidney’s blood pressure regulating abilities, leading to higher blood pressure [25]. This increased blood pressure causes small changes in blood flow, which exposes other sensitive capillaries to additional damage. The elevated blood pressure can also affect the delicate insulin secreting areas of the pancreas, leading to higher blood sugar [26]. In this way, the combination of elevated blood pressure and high blood sugar is a self−reinforcing loop in which both diseases tend to worsen over time.

Individuals with IFG and/or IGT are referred to as having prediabetes, which reflects a high risk for developing diabetes and cardiovascular disease [27]. One of the major findings of the present study is to detect the risk factors of prediabetes: urban area, high blood pressure, body fat percentage, and waist–hip ratio were associated with IGT; urban area, high blood pressure, and alcohol consumption were associated with IFG. These risk factors have been reported in many studies, and their different influences vary among countries [3,5,6,28,29]. In terms of the relationship between alcohol consumption and hyperglycemia levels, there has not been reported previously in Vietnam although using rice wine is common seen in the rural areas. In this study, there was no association of alcohol consumption with diabetes and IGT. Interestingly, in line with studies in Korea and China [30,31], we reported that subjects with one drink/month or more were 2.2–fold more likely to have IFG than those without drinking alcohol in the models adjusted for potential confounding factors. However, it is inconsistent with a prospective study in Japanese men office workers [32]. In the context of further studies needed to clarify the controversial effect of alcohol consumption on hyperglycemia, our observation in Vietnamese population supports the call by the World Health Organization to implement evidence−based strategies to reduce harmful use of alcohol [33].

The present study has found several important findings for public health policy makers in prevention of diabetes in Vietnam. First, the newly diagnosed diabetic cases occupied 75% of the total diabetic subjects, and this rate in the year 2011 was more frequent than those in a previous report of 9 years ago [3], suggesting the major part of diabetic residents in rural areas without knowing this condition. It highlights the urgent need for greater public awareness on risk factors for hyperglycemia status and strengthening of diabetes–related health services to detect, prevent, and treat early individuals with diabetes. In addition, because Ha Nam province is thought to be a typical rural province in Red River Delta Region with a population about 3,727,000 adults aged 40–64 years [8], we could estimate 137,900 diabetic residents, 100,600 undiagnosed patients, and 547,900 prediabetes cases in the region. Second, our study indicates that the single FPG test may lead to underestimate about 40 % of diabetic cases in rural population, suggesting OGTT method should be the appropriate way to screening diabetes and prediabetes in the communities. Moreover, the age–standardized prevalence of IFG defined by OGTT test with intravenous blood in our study (8.8%) was 4.6–fold higher than that in the 2002 national survey of diabetes in the plains region (1.9%), in which OGTT test with capillary blood was used, suggesting the hypothesis that the use of such OGTT test with capillary blood may underestimate the prevalence of IFG. Further study needs to confirm the hypothesis before recommendation of this method for screening IFG in Vietnamese populations. Third, the BMI means (SD) of the diabetes group were 22.4 (3.4) in men and 22.7 (3.5) in women, in agreement with the observation that the risk of diabetes starts at a lower BMI for Asians than for Europeans [28], supporting the use of recommendations to classify overweight and obesity for Asian and Pacific Island populations, which corresponds to the BMI cutoffs of 23 and 25 kg/m^2^[10,11].

The present findings must be interpreted in the context of several potential limitations. First, the study was limited by its cross–sectional nature, and this does not allow for conclusions of the causal relationships. Second, data on physical activities, food intake, and blood lipids were not used in these findings to evaluate a potential effect of these variables in our results. Next, blood samples were collected and centrifuged immediately in the morning after a participant had fasted for at least 8h prior to the clinic visit. Aliquots of plasma were stored at 2–8^o^ C in iceboxes and then transported into the central laboratory for analysis within 6 hours. However, although this process we used can minimize the preanalytical impact on detecting diabetes, it may cause about 2% underestimate of prediabetes prevalence [34]. Lastly, our sample was a representative sample for a rural province in the Red River Delta region, the extrapolation for other geographical regions in Vietnam (Mountainous Northwest and Northeast, North Central Coast, South Central Coast, Central Highlands, Southeast, and Mekong River Delta) should be taken into account. It is essential to conduct a national survey on diabetes to evaluate the burden of the disease in different geographic regions.

## Conclusions

In summary, the study showed that the hyperglycemia status in rural Vietnameses was less severe than that in urban residents. Family history of diabetes, blood pressure, obesity−related measures, residence, and alcohol consumption were positively associated with hyperglycemia levels. There is an urgent need for greater public awareness on risk factors for hyperglycemia status and strengthening of diabetes–related health services to early detect, prevent, and treat individuals with diabetes.

## Abbreviations

IFG: Impaired fasting glucose; IGT: Impaired glucose tolerance; FPG: Fasting plasma glucose level; OGTT: Oral glucose tolerance test; BMI: Body mass index; WC: Waist circumference; HC: Hip circumference; WHR: Waist–hip ratio; SBP: Systolic blood pressure; DBP: Diastolic blood pressure.

## Competing interests

The authors declare that they have no competing interests.

## Authors’ contributions

TQB: Conceptualization of the study, study design, proposal writing, data collection, data analysis, discussion and editing of the final draft for publication. BTN, DDT: Conceptualization of the study, study design, data collection, data analysis, discussion and editing of the final draft for publication. PTP: Conceptualization of the study, study design, data collection, and editing of the final draft for publication. PVT, TKL, and DVT: Study design, data collection, data analysis, and discussion. All authors approved the final draft of this article prior to submission. All authors read and approved the final manuscript.

## Pre-publication history

The pre-publication history for this paper can be accessed here:

http://www.biomedcentral.com/1471-2458/12/939/prepub

## Supplementary Material

Additional file 1 Associated factors of IFG, IGT, IFG−IGT and diabetes mellitus in univariate logistic regression analysis. Click here for file

## References

[B1] Shaw JE Sicree RA Zimmet PZ Global estimates of the prevalence of diabetes for 2010 and 2030 Diabetes Res Clin Pract 2010 87 4 14 10.1016/j.diabres.2009.10.007 19896746

[B2] Quoc PS Charles MA Cuong NH Lieu LH Tuan NA Thomas M Balkau B Simon D Blood glucose distribution and prevalence of diabetes in Hanoi (Vietnam) Am J Epidemiol 1994 139 713 722 8166132 10.1093/oxfordjournals.aje.a117061

[B3] Binh VT Uoc KH Epidemiology of diabetes in Vietnam: Treatment methods and preventive measures 2005 Hanoi: Medical Publisher

[B4] Trach MT Toan DTB Binh DTT Thang HQ Basic epidemiology survey on diabetes mellitus in urban areas of Ho Chi Minh City Pharmaceutical and Medical Magazine of Ho Chi Minh City 1994 2 171 174

[B5] Son LNTD Kusama K Hung NTK Loan TTH Chuyen NV Kunii D Sakai T Yamamoto S Prevalence and risk factors for Diabetes in Ho Chi Minh City, Vietnam Diabetic Med 2004 21 371 376 10.1111/j.1464-5491.2004.01159.x 15049941

[B6] Ta MT Nguyen KT Nguyen ND Campbell LV Nguyen TV Identification of undiagnosed type 2 diabetes by systolic blood pressure and waist–to–hip ratio Diabetologia 2010 53 2139 2146 10.1007/s00125-010-1841-6 20596691

[B7] Khoi HH Problems of nutrition in transition period. Problems of Nutrition in Transition Period in Vietnam 1996 Hanoi: Medical Publishing House 153 226

[B8] General Statistics Office The 2009 Vietnam Population and Housing Census: Completed results 2012 Hanoi: Statistical Publishing House Available from www.gso.gov.vn

[B9] World Health Organization Definition and diagnosis of diabetes mellitus and intermediate hyperglycemia: Report of a WHO consultation 2006 Geneva: World Health Organization

[B10] WHO Expert Consultation Appropriate body–mass index for Asian populations and its implications for policy and intervention strategies Lancet 2004 363 157 163 14726171 10.1016/S0140-6736(03)15268-3

[B11] International Obesity Task Force (on behalf of the Steering Committee) The Asia–Pacific Perspective: Redefining obesity and its treatment 2002 Sydney: Western Pacific Region. Health Communications Australia

[B12] Jonas JB Panda Jonas S Diabetes mellitus in rural India Epidemiology 2010 21 754 755 10.1097/EDE.0b013e3181e66201 20699685

[B13] Zhang YH Ma WJ Thomas GN Xu YJ Lao XQ Xu XJ Song XL Xu HF Cai QM Xia L Nie SP Deng HH Yu IT Diabetes and pre–diabetes as determined by glycated haemoglobin a1c and glucose levels in a developing southern Chinese population PLoS One 2012 7 e37260 10.1371/journal.pone.0037260 22615957 PMC3352895

[B14] Aekplakorn W Chariyalertsak S Kessomboon P Sangthong R Inthawong R Putwatana P Taneepanichskul S Thai National Health Examination Survey IV Study Group Prevalence and management of diabetes and metabolic risk factors in Thai adults: the Thai National Health Examination Survey IV, 2009 Diabetes Care 2011 34 1980 1985 10.2337/dc11-0099 21816976 PMC3161276

[B15] Sekikawa A Eguchi H Tominaga M Igarashi K Abe T Manaka H Sasaki H Fukuyama H Kato T Kiyohara Y Fujishima M Prevalence of type 2 diabetes mellitus and impaired glucose tolerance in a rural area of Japan: the Funagata Diabetes Study J Diabetes Complications 2006 14 78 83 10959069 10.1016/s1056-8727(00)00074-x

[B16] Kim DJ The epidemiology of diabetes in Korea Diabetes Metab J 2011 35 303 308 10.4093/dmj.2011.35.4.303 21977448 PMC3178689

[B17] Popkin BM Horton SH Kim S The Nutrition Transition and Prevention of Diet–Related Diseases in Asia and Pacific Food Nutri Bullet 2001 22 1 58

[B18] James C Bullard KM Rolka DB Geiss LS Williams DE Cowie CC Albright A Gregg EW Implications of alternative definitions of prediabetes for prevalence in U.S. adults Diabetes Care 2011 34 387 391 10.2337/dc10-1314 21270196 PMC3024354

[B19] Zhang YH Ma WJ Thomas GN Xu YJ Lao XQ Diabetes and prediabetes as determined by glycated haemoglobin A1c and glucose levels in a developing southern Chinese population PLoS One 2012 7 e37260 10.1371/journal.pone.0037260 22615957 PMC3352895

[B20] Tabák AG Herder C Rathmann W Brunner EJ Kivimäki M Prediabetes: a high-risk state for diabetes development Lancet 2012 379 2279 2290 10.1016/S0140-6736(12)60283-9 22683128 PMC3891203

[B21] Govindarajan G Sowers JR Stump CS Hypertension and diabetes mellitus European Cardiology 2006 2 1 7

[B22] Roccella EJ Sowers JR Cutler JA Donato K Eastman RC Falkner B Horan MJ Hsueh WA Hyman B Reed JW Savage PJ Siwek J Tuck ML Weinberger MH Williams G National High Blood Pressure Education Program Working Group report on hypertension in diabetes Hypertension 1994 23 145 158 8307622

[B23] Mullican DR Lorenzo C Haffner SM Is prehypertension a risk factor for the development of Type2 diabetes? Diabetes Care 2009 32 1870 1872 10.2337/dc09-0328 19628813 PMC2752916

[B24] Conen D Ridker PM Mora S Buring JE Glynn RJ Blood pressure and risk of developing type 2 diabetes mellitus: the Women’s Health Study Eur Heart J 2007 28 2937 2943 10.1093/eurheartj/ehm400 17925342

[B25] Cruickshank K Riste L Anderson SG Wright JS Dunn G Gosling RG Aortic pulse-wave velocity and its relationship to mortality in diabetes and glucose intolerance: an integrated index of vascular function? Circulation 2002 106 2085 2090 10.1161/01.CIR.0000033824.02722.F7 12379578

[B26] Dokken BB The pathophysiology of cardiovascular disease and diabetes: beyond blood pressure and lipids Diabetes Spectrum 2008 21 160 165 10.2337/diaspect.21.3.160

[B27] American Diabetes Association Diagnosis and classification of diabetes mellitus Diabetes Care 2005 28 S37 S42 15618111 10.2337/diacare.28.suppl_1.s37

[B28] Chan JC Malik V Jia W Kadowaki T Yajnik CS Yoon KH Hu FB Diabetes in Asia: epidemiology, risk factors, and pathophysiology JAMA 2009 301 2129 2140 10.1001/jama.2009.726 19470990

[B29] Weber MB Oza Frank R Staimez LR Ali MK Narayan KM Type 2 Diabetes in Asians: Prevalence, Risk Factors, and Effectiveness of Behavioral Intervention at Individual and Population Levels Annu Rev Nutr 2012 10.1146/annurev-nutr-071811-150630 22524185

[B30] Kim SM Lee JS Lee J Na JK Han JH Yoon DK Baik SH Choi DS Choi KM Prevalence of diabetes and impaired fasting glucose in Korea: Korean National Health and Nutrition Survey 2001 Diabetes Care 2006 29 226 231 10.2337/diacare.29.02.06.dc05-0481 16443864

[B31] Liu C Yu Z Li H Wang J Sun L Qi Q Lin X Associations of alcohol consumption with diabetes mellitus and impaired fasting glycemia among middle-aged and elderly Chinese BMC Publ Health 2010 10 713 10.1186/1471-2458-10-713 PMC2998495 21092093

[B32] Nakanishi N Suzuki K Tatara K Alcohol consumption and risk for development of impaired fasting glucose or type 2 diabetes in middle-aged Japanese men Diabetes Care 2003 26 48 54 10.2337/diacare.26.1.48 12502657

[B33] Parry CD Patra J Rehm J Alcohol consumption and non-communicable diseases: epidemiology and policy implications Addiction 2011 106 1718 1724 10.1111/j.1360-0443.2011.03605.x 21819471 PMC3174337

[B34] Stahl M Jorgensen LG Hyltoft PP Brandslund I de Fine Olivarius N Borch-Johnsen K Optimization of preanalytical conditions and analysis of plasma glucose. 1. Impact of the new WHO and ADA recommendations on diagnosis of diabetes mellitus Scand J Clin Lab Invest 2001 61 169 179 10.1080/003655101300133612 11386604

