# Supplementary material for: Prevalence and correlates of hyperglycemia in a rural population, Vietnam: implications from a cross–sectional study
Source: BMC Public Health. 2012 Nov 1;12:939. doi: 10.1186/1471-2458-12-939 (PMC3548760; doi:10.1186/1471-2458-12-939)
Supplement: Additional file 1 — Associated factors of IFG, IGT, IFG−IGT and diabetes mellitus in univariate logistic regression analysis. [file 1471-2458-12-939-S1.doc]

**Additional table 1. *Associated factors of IFG, IGT, IFG−IGT and diabetes mellitus in univariate logistic regression analysis***

|  | IFG (*n* = 248) |  | . | IGT (*n* = 120) |  | . | IFG−IGT (*n* = 43) | | . | Diabetes mellitus (*n* = 100) | |
| --- | --- | --- | --- | --- | --- | --- | --- | --- | --- | --- | --- |
|  | OR (95%CI) | *P* |  | OR (95%CI) | *P* |  | OR (95%CI) | *P* |  | OR | *P* |
| Sex |  |  |  |  |  |  |  |  |  |  |  |
| Femal | 1 |  |  | 1 |  |  | 1 |  |  | 1 |  |
| Male | 1.16 (0.88–1.52) | 0.300 |  | 1.20 (0.82–1.75) | 0.354 |  | 0.83 (0.43–1.61) | 0.588 |  | 1.57 (1.05–2.36) | **0.028** |
| Age (year) | 1.04 (1.01–1.06) | **0.001** |  | 1.04 (1.01–1.07) | **0.006** |  | 1.02 (0.98–1.07) | 0.383 |  | 1.07 (1.03–1.10) | **<0.0001** |
| Blood pressure (mmHg) |  |  |  |  |  |  |  |  |  |  |  |
| Normal | 1 |  |  | 1 |  |  | 1 |  |  | 1 |  |
| High | 1.81 (1.38–2.37) | **< 0.0001** |  | 2.17 (1.49–3.18) | **< 0.0001** |  | 1.78 (0.96–3.31) | 0.067 |  | 2.51 (1.67–3.78) | **<0.0001** |
| Nutrition status |  |  |  |  | 0.334 |  |  |  |  |  |  |
| Normal | 1 |  |  | 1 |  |  | 1 |  |  | 1 |  |
| Overweight | 1.31 (0.92–1.86) | 0.132 |  | 1.23 (0.75–2.01) | 0.418 |  | 1.16 (0.52–2.57) | 0.722 |  | 1.66 (0.97–2.84) | 0.064 |
| Obesity | 1.00 (0.60–1.65) | 0.985 |  | 1.99 (1.17–3.38) | **0.011** |  | 1.63 (0.66–4.0) | 0.286 |  | 3.28 (1.92–5.61) | **< 0.0001** |
| Underweight | 1.27 (0.88–1.84) | 0.195 |  | 0.49 (0.23–1.02) | 0.056 |  | 0.32 (0.07–1.33) | 0.116 |  | 0.99 (0.51–1.94) | 0.986 |
| Weight (kg) | 1.00 (0.99–1.02) | 0.772 |  | 1.03 (1.01–1.05) | **0.018** |  | 1.02 (0.98–1.06) | 0.294 |  | 1.06 (1.04–1.09) | **< 0.0001** |
| BMI (kg/m^2^) | 0.99 (0.95–1.05) | 0.855 |  | 1.13 (1.06–1.20) | **< 0.0001** |  | 1.14 (1.03–1.27) | **0.016** |  | 1.19 (1.11–1.27) | **< 0.0001** |
| Body fat (%) | 1.00 (0.98–1.02) | 0.800 |  | 1.05 (1.01–1.08) | **0.004** |  | 1.05 (1.00–1.10) | 0.062 |  | 1.05 (1.01–1.08) | **0.008** |
| Waist circumference (cm) | 1.01 (0.99–1.03) | 0.285 |  | 1.05 (1.03–1.07) | **< 0.0001** |  | 1.02 (0.99–1.06) | 0.237 |  | 1.09 (1.07–1.12) | **< 0.0001** |
| Hip circumference (cm) | 0.98 (0.96–1.01) | 0.161 |  | 1.03 (0.99–1.06) | 0.132 |  | 1.02 (0.97–1.08) | 0.498 |  | 1.08 (1.04–1.12) | **< 0.0001** |
| Waist-hip ratio (SD=0.07) | 1.29 (1.11–1.50) | **0.001** |  | 1.56 (1.30–1.87) | **< 0.0001** |  | 1.24 (0.88–1.75) | 0.225 |  | 1.80 (1.50–2.16) | **< 0.0001** |
| Abdominal obesity |  |  |  |  |  |  |  |  |  |  |  |
| No | 1 |  |  | 1 |  |  | 1 |  |  | 1 |  |
| Yes | 1.25 (0.95–1.64) | 0.113 |  | 1.98 (1.37–2.87) | **< 0.0001** |  | 0.95 (0.49–1.83) | 0.878 |  | 2.91 (1.94–4.36) | **< 0.0001** |
| Family history of diabetes | |  |  |  |  |  |  |  |  |  |  |
| No | 1 |  |  | 1 |  |  | 1 |  |  | 1 |  |
| Yes | 1.39 (0.73–2.66) | 0.319 |  | 2.14 (1.01–4.56) | **0.048** |  | 2.25 (0.68–7.44) | 0.185 |  | 4.09 (2.14–7.81) | **<0.0001** |
| Marrital status |  |  |  |  |  |  |  |  |  |  |  |
| Married | 1 |  |  | 1 |  |  | 1 |  |  | 1 |  |
| Never | 1.88 (0.82–4.28) | 0.135 |  | 1.63 (0.49–5.39) | 0.423 |  | 6.58 (2.22–19.5) | **0.001** |  | 2.69 (0.94–7.75) | 0.066 |
| Widowed | 1.32 (0.78–2.24) | 0.297 |  | 0.95 (0.41–2.20) | 0.900 |  | 1.43 (0.44–4.73) | 0.553 |  | 1.37 (0.62–3.02) | 0.437 |
| Others | 0.80 (0.25–2.64) | 0.719 |  | 1.09 (0.26–4.58) | 0.910 |  | – | – |  | 0.67 (0.09–4.97) | 0.698 |
| Education level |  |  |  |  |  |  |  |  |  |  |  |
| Elementary | 1 |  |  | 1 |  |  | 1 |  |  | 1 |  |
| Intermediate | 1.34 (0.82–2.17) | 0.242 |  | 0.88 (0.48–1.62) | 0.678 |  | 0.32 (0.14–0.73) | 0.006 |  | 1.03 (0.48–2.19) | 0.947 |
| Secondary | 1.08 (0.59–1.96) | 0.809 |  | 1.12 (0.54–2.33) | 0.752 |  | 0.34 (0.10–1.13) | 0.077 |  | 1.73 (0.74–4.05) | 0.207 |
| Post–secondary | 1.15 (0.65–2.05) | 0.634 |  | 0.89 (0.42–1.86) | 0.747 |  | 0.90 (0.37–2.18) | 0.820 |  | 1.86 (0.81–4.26) | 0.141 |
| Residence |  |  |  |  |  |  |  |  |  |  |  |
| Rural | 1 |  |  | 1 |  |  | 1 |  |  | 1 |  |
| Urban | 2.77 (1.76–4.34) | **< 0.0001** |  | 2.99 (1.65–5.42) | **< 0.0001** |  | 4.40 (1.91–10.2) | **0.001** |  | 2.24 (1.10–4.58) | **0.027** |
| Heavy occupation |  |  |  |  |  |  |  |  |  |  |  |
| Yes | 1 |  |  | 1 |  |  | 1 |  |  | 1 |  |
| No | 0.99 (0.71–1.37) | 0.927 |  | 1.49 (0.98–2.27) | 0.061 |  | 1.59 (0.81–3.12) | 0.179 |  | 2.11 (1.38–3.24) | **0.001** |
| Income level |  |  |  |  |  |  |  |  |  |  |  |
| < 25 percentiles | 1 |  |  | 1 |  |  | 1 |  |  | 1 | 1 |
| 25–<50 percentiles | 1.06 (0.73–1.55) | 0.749 |  | 1.65 (1.00–2.71) | 0.051 |  | 1.33 (0.56–3.17) | 0.526 |  | 0.84 (0.47–1.50) | 0.560 |
| 50–75< percentiles | 1.11 (0.76–1.62) | 0.607 |  | 0.82 (0.45–1.49) | 0.517 |  | 1.42 (0.60–3.41) | 0.427 |  | 0.95 (0.53–1.68) | 0.846 |
| ≥75 percentiles | 1.24 (0.86–1.80) | 0.249 |  | 1.26 (0.74–2.16) | 0.391 |  | 1.18 (0.48–2.92) | 0.724 |  | 1.18 (0.69–2.03) | 0.545 |
| Alcohol consumption |  |  |  |  |  |  |  |  |  |  |  |
| None | 1 |  |  | 1 |  |  | 1 |  |  | 1 |  |
| <1 drink/mo | 1.11 (0.63–1.93) | 0.728 |  | 1.00 (0.45–2.20) | 0.990 |  | 1.02 (0.31–3.40) | 0.970 |  | 1.04 (0.44–2.45) | 0.927 |
| ≥ 1 drink/mo to < 1 drink/wk | 1.59 (0.95–2.66) | 0.076 |  | 1.46 (0.71–2.98) | 0.304 |  | 1.94 (0.74–5.09) | 0.178 |  | 1.38 (0.62–3.09) | 0.431 |
| 1 drink/wk to ≤ 1 drink/d | 1.59 (1.11–2.28) | **0.011** |  | 0.96 (0.53–1.72) | 0.885 |  | 0.66 (0.23–1.88) | 0.433 |  | 0.84 (0.42–1.65) | 0.603 |
| ≥ 2 drink/d | 1.46 (0.96–2.22) | 0.078 |  | 1.69 (0.99–2.89) | 0.055 |  | 0.23 (0.03–1.66) | 0.144 |  | 2.06 (1.19–3.56) | **0.010** |
| Smoking |  |  |  |  |  |  |  |  |  |  |  |
| None | 1 |  |  | 1 |  |  | 1 |  |  | 1 |  |
| Current smoker | 1.02 (0.72–1.46) | 0.896 |  | 1.01 (0.60–1.67) | 0.985 |  | 0.56 (0.20–1.60) | 0.280 |  | 1.12 (0.65–1.94) | 0.673 |
| Ex–smoker | 1.20 (0.78–1.84) | 0.406 |  | 1.51 (0.87–2.62) | 0.147 |  | 1.75 (0.76–4.02) | 0.187 |  | 1.77 (0.99–3.15) | 0.054 |
| Watching TV time/day |  |  |  |  |  |  |  |  |  |  |  |
| ≤ 3 hours | 1 |  |  | 1 |  |  | 1 |  |  | 1 |  |
| > 3 hours | 0.93 (0.49–1.75) | 0.812 |  | 1.05 (0.45–2.44) | 0.910 |  | 1.50 (0.46–4.91) | 0.507 |  | 2.47 (1.28–4.75) | **0.007** |
| Siesta time/day |  |  |  |  |  |  |  |  |  |  |  |
| None | 1 |  |  | 1 |  |  | 1 |  |  | 1 |  |
| <30 min | 1.27 (0.82–1.97) | 0.286 |  | 0.86 (0.49–1.54) | 0.617 |  | 2.05 (0.59–7.11) | 0.261 |  | 1.74 (0.80–3.80) | 0.165 |
| 30–<60 min | 1.11 (0.69–1.80) | 0.667 |  | 0.97 (0.52–1.80) | 0.923 |  | 1.57 (0.40–6.11) | 0.517 |  | 1.68 (0.73–3.86) | 0.222 |
| 60–<90 min | 1.35 (0.84–2.17) | 0.208 |  | 0.99 (0.53–1.85) | 0.979 |  | 2.62 (0.72–9.46) | 0.142 |  | 1.96 (0.86–4.47) | 0.108 |
| ≥ 90 min | 1.34 (0.71–2.51) | 0.367 |  | 1.14 (0.50–2.60) | 0.755 |  | 4.56 (1.13–18.5) | **0.034** |  | 3.42 (1.37–8.55) | **0.009** |
| Sleeping time/day |  |  |  |  |  |  |  |  |  |  |  |
| ≥ 8 hours | 1 |  |  | 1 |  |  | 1 |  |  | 1 |  |
| 6–7 hours | 0.97 (0.71–1.34) | 0.867 |  | 1.32 (0.81–2.16) | 0.261 |  | 0.62 (0.31–1.21) | 0.164 |  | 1.35 (0.78–2.35) | 0.286 |
| < 6 hours | 1.07 (0.70–1.65) | 0.754 |  | 1.59 (0.86–2.93) | 0.140 |  | 0.59 (0.21–1.67) | 0.322 |  | 2.06 (1.07–3.95) | **0.031** |
| Sitting time/day |  |  |  |  |  |  |  |  |  |  |  |
| ≤ 4 hours | 1 |  |  | 1 |  |  | 1 |  |  | 1 |  |
| > 4 hours | 0.91 (0.68–1.21) | 0.513 |  | 0.92 (0.62–1.37) | 0.672 |  | 1.63 (0.89–2.99) | 0.116 |  | 1.55 (1.04–2.33) | **0.034** |

NGT, normal glucose tolerance; IFG, isolated impaired fasting glucose; IGT, isolated impaired glucose tolerance; IGF−IGT, combined IFG and IGT.

Abdominal obesity is recored if waist-hip ratio ≥ 0.90 in males or ≥ 0.85 in females. High blood pressure is defined as systolic blood pressure ≥ 130 mmHg and/or a diastolic blood pressure ≥ 85 mmHg. Boldface type indicates statistical significance.
